# Risk Factors of Rheumatic Heart Disease in a Children’s Hospital in Khartoum: A Case-Control Study

**DOI:** 10.7759/cureus.69786

**Published:** 2024-09-20

**Authors:** Imtinan Yousif, Safaa Fadlelmoula, Eithar M Ali, Shima Alhindi, Doaa Mohammadat, Ibrahim H Elkhidir, Sulafa Ali

**Affiliations:** 1 Ophthalmology, University of Khartoum, Khartoum, SDN; 2 Ophthalmology, Sudan Medical Specialization Board, Khartoum, SDN; 3 Community Medicine, University of Khartoum, Khartoum, SDN; 4 Medicine, University of Khartoum, Khartoum, SDN; 5 Obstetrics and Gynecology, Letterkenny University Hospital, Letterkenny, IRL; 6 Research, Sustainable Development Response Organization (SUDRO), Mogadishu, SOM; 7 Emergency Multidisciplinary Unit, Letterkenny University Hospital, Letterkenny, IRL; 8 Pediatric Cardiology, Khartoum University, Khartoum, SDN

**Keywords:** acute rheumatic fever, arf, case-control study, rhd, rheumatic heart disease, risk factors, sudan

## Abstract

Background: Rheumatic heart disease (RHD) is the most prevalent acquired cardiac illness in Sudan, arising as a complication of acute rheumatic fever (ARF). Additionally, Sudan exhibited a wide diversity in the distribution of RHD. The echocardiographic screening revealed 3/1000 cases in one region (Khartoum), while in another region (Nort Kordofan), it revealed 61/1000 cases. Consequently, further research is warranted to shed light on this disease, particularly its risk factors. There is a lack of research on the risk factors in Sudan. The objective of this study is to evaluate Sudan's RHD risk factors.

Methods: This case-control study was conducted at Jafar Ibn Auf Children's Hospital from August to December 2016. A questionnaire was used to study RHD risk factors, including socioeconomic status, maternal education and employment, housing conditions, residence, and history of sore throat and ARF. Comparison between cases and controls was estimated using conditional logistic regression with 95% confidence intervals (CI).

Results: A total of 78 participants, including 39 established RHD cases and 39 age- and sex-matched normal controls, were recruited. The age in years for cases and controls was 11.1 +/- 2.9 and 11+/- 2.9, respectively. In the univariate analysis, only the history of ARF was significantly associated with RHD (odds ratio (OR) 60.8, 95% CI 10.3-356). There was a trend toward increased risk of RHD in association with a history of sore throat in 69.2% of the patients and origin from western states in 41% of the patients, but this did not reach statistical significance.

Conclusions: ARF history and a six-month history of throat infections are linked to RHD. Mothers do not have a correlation with RHD, but a correlation exists when there is a connection between a history of ARF and a recent throat infection (effect modification). Furthermore, an association was found between throat infections and living in a western state. Consequently, it is imperative to adopt RHD prevention, particularly the prevention and treatment of both ARF and throat infections.

## Introduction

Approximately 30 million people worldwide are believed to be afflicted by rheumatic heart disease (RHD), which was estimated to have caused 305,000 deaths and 11.5 million years of life lost due to disability in 2015. Moreover, RHD is considered one of the major health issues in low- and middle-income countries (LMICs). The worst-affected regions include Africa, Southeast Asia, and the Western Pacific, which accounted for 84% of all prevalent cases and 80% of all anticipated fatalities from RHD in 2015 [1]. The main feature of RHD is irreversible damage to the heart's valves, which arises as a major aftereffect of acute rheumatic fever (ARF), an inflammatory response to a Group A beta-hemolytic streptococcus (GAS) bacterial infection [2]. The most typical manifestation of RHD in young individuals is mitral regurgitation. However, RHD is the most frequent cause of mitral stenosis globally [3]. Regarding risk factors of RHD, several socioeconomic issues can influence the occurrence of RHD, such as sharing others in bed and low education of mothers [4]. In addition, genetic factors play a role in the development of RHD [5].

The most prevalent acquired heart disease in this demographic of socially and economically disadvantaged people is RHD [6-10]. South Asia, Oceania, and central sub-Saharan Africa have the highest age-standardized rates of RHD death and prevalence. Despite this, the disease is endemic in most low- and middle-income nations [11]. RHD is the most common cause of cardiac morbidity and mortality in Sudan [12]. Therefore, this project aimed to delineate the risk factors for RHD in Sudanese children.

## Materials and methods

Study type, population, and sampling

This study focused on children diagnosed with RHD based on echocardiographic findings for the case group. Control participants were selected from other clinics and matched by age and sex to the case group. The study was conducted over four months to achieve the target sample size. Critically ill patients, including those in the emergency room requiring immediate stabilization, were excluded from the study.

In this study, we employed non-probability sequential sampling primarily due to practical constraints such as limited time and resources and the need to efficiently access a relevant population of RHD patients and matched controls. Given the rarity of RHD, this method allowed us to gather sufficient data from available cases and controls within the study's four-month timeframe. Additionally, non-probability sequential sampling facilitated the matching of controls by age and sex with the case group, ensuring that our sample met the study's requirements for comparability. The sample size was calculated using the formula n=2(z²pq/d²), where n = sample size, z = standard normal z score (1.96 for 95% confidence interval (CI)), p = prevalence of RHD in Sudan, which is 0.03, q = 1-p, and d = desired margin of error. The sample was 78 (39 cases and 39 controls). The average age of the participants was 11.1 ± 3 years, and the majority (61.5%) were females.

Data collection

The participants were interviewed using a structured, pre-tested questionnaire. The questionnaire captured sociodemographic details, including maternal information, home environment, and history of ARF and throat infections in the past six months.

Data management and analysis

Data were entered into Microsoft Excel (Microsoft Corporation, Redmond, Washington) for initial cleaning and auditing before being transferred to the IBM SPSS Statistics for Windows, Version 26 (released 2019; IBM Corp., Armonk, New York) for analysis. Preliminary statistics included generating frequency tables. For inferential analysis, the Student's t-test was applied to continuous data, following normality checks using the Kolmogorov-Smirnov and Shapiro-Wilk tests. A p-value ≤ 0.05 was considered statistically significant.

## Results

Sociodemographic factors

There is no significant difference across the two groups (cases and controls) concerning family size, persons per room, and the monthly family income. Furthermore, our study could not detect a significant association between residence (rural versus urban areas) and geographical distribution with RHD (Table 1).

**Table 1:** Sociodemographic variables among RHD cases and controls (n = 78) SDG: Sudanese pounds

Variable	RHD Cases N	Controls N	Unadjusted Odds Ratio
Mean age	11.1 (SD: 2.9) years	11 (SD: 2.9) years	-
Gender			
Female	24 (61.5%)	24 (61.5%)	-
Male	15 (38.5%)	15 (38.5%)	-
Family size			OR 0.4 (95% CI 0.1–1.3)
≤ 8	22 (56.4%)	27 (69.2%)	-
>8	17 (43.6%)	12 (30.8%)	-
Persons per room			OR 1.6 (95% CI 0.6–4.4)
≤3	22 (56.4%)	20 (51.3%)	-
>3	17 (43.6%)	19 (48.7%)	-
Family income/month ≤ 1000 SDG (60 $)	19 (48.7%)	18 (46.2%)	OR 1.1 (95% CI 0.4–2.8)
>1000 SDG (60 $)	20 (51.3%)	21 (53.8%)	-
Residence			OR 0.4 (95% CI 0.1–1.2)
Rural	20 (51.3%)	14 (35.9%)	-
Urban	19 (48.7%)	25 (64.1%)	-
Geographic distribution			OR 2.6 (95% CI 0.3–19)
Khartoum state	6 (15.3%)	3 (7.7%)	-
Blue Nile states	15 (38.5%)	17 (43.6%)	-
Western states	16 (41%)	12 (30.7%)	-
Northern states	1 (2.6%)	4 (10.3%)	-
Eastern states	1 (2.6%)	3 (7.7%)	-

Educational and occupational status of mothers

Regarding mother status, there was a trend toward an association between having an unemployed mother or a mother with only a primary level of education and RHD; however, this did not reach statistical significance (Table 2).

**Table 2:** Educational and occupational status of the mothers OR: odds ratio; CI: confidence interval; a logistic regression method

Characteristics	Cases (n = 39)	Controls (n = 39)	Odds Ratio
Education	-	-	OR 2.8 (95% CI 0.8–9.1)
Primary or less	34 (87.2%)	27 (69.2%)	-
Secondary and above	5 (12.8%)	12 (30.8%)	-
Occupation	-	-	OR 1.7 (95% CI 0.5–6)
Unemployed	34 (87.2%)	30 (76.9%)	-
Employed	5 (12.8%)	9 (23.1%)	-

History of ARF and throat infection in the past six months

The proportion of participants with a history of ARF was significantly higher among the RHD group (OR* 60.8 (95% CI 10.3-356)). However, although 69.2% of the patients had a positive history of throat infection, this did not reach statistical significance (Table 3).

**Table 3:** History of ARF and throat infection in the past six months among the study participants OR* statistically significant ARF: acute rheumatic fever; OR: odds ratio; CI: confidence interval; a logistic regression method

Focused History	Cases (n = 39)	Controls (n = 39)	Statistical Test^a^
ARF	-	-	OR* 60.8 (95% CI 10.3–356)
Yes	32 (82.1%)	2 (5.1%)	-
No	7 (17.9%)	37 (94.9%)	-
Throat infections in the past six months	-	-	OR 2.7 (95% CI 0.5–14)
Yes	27 (69.2%)	9 (23.1%)	-
No	12 (30.8%)	30 (76.9%)	-

Stratification and subgroup analysis

When investigating the association between a history of throat infection in the last six months and RHD, it was only significant among patients whose mothers were unemployed (effect modification) (Table 4).

**Table 4:** Stratification and subgroup analysis RHD: rheumatic heart disease

Risk Estimate				
Occupation		Value	95% Confidence interval	
			Lower	Upper
Unemployed mother	The odds ratio for a history of throat infection in the past six months (with history/without history)	7.639	2.514	23.212
	For the cohort who suffer from RHD = have the disease	2.609	1.456	4.676
	For the cohort who suffer from RHD = do not have the disease	.342	.179	.650
	N of valid cases	64	-	-
Employed mother	The odds ratio for a history of throat infection in the past six months (with history/without history)	5.333	.343	82.827
	For the cohort who suffer from RHD = have the disease	2.444	.698	8.563
	For the cohort who suffer from RHD = do not have the disease	.458	.089	2.364
	N of valid cases	14	-	-
Total	The odds ratio for a history of throat infection in the past six months (with history/without history)	7.500	2.735	20.563
	For the cohort who suffer from RHD = have the disease	2.625	1.570	4.389
	For the cohort who suffer from RHD = do not have the disease	.350	.193	.636
	N of valid cases	78	-	-

## Discussion

Despite the findings by Qurashi in Saudi Arabia (2009) [13] and Breda et al. in Italy (2012) [14], which identified a significant association between socioeconomic class and an increased risk of RHD, our study did not observe a similar relationship. This lack of association between cases and controls may be attributed to the fact that most individuals in Sudan are informal workers with irregular or no fixed monthly income. This may potentially underestimate their actual monthly income. Furthermore, in poor communities, wealth is typically low, and there were no large differences in social classes to affect risk factors of the disease significantly. This is in line with the findings of Steer et al., who found that in developing nations, a threshold level where better socioeconomic status is associated with a lower prevalence of RHD has not been achieved [15].

Regarding overcrowding, research conducted by Okello et al. in Uganda (2012), Webb and Wilson in New Zealand (2013), and Riaz et al. in Bangladesh (2013) found a strong association between RHD and overcrowding [16-18]. However, in India, Joseph et al. (2013) found that overcrowding is not associated with ARF/RHD [19]. The latter's findings are consistent with our finding that overcrowding is not directly associated with RHD, possibly due to the masking factor of Sudanese houses being large landmasses and the fact that they stay most of the time in their house yards and sleep there.

As GAS pharyngitis is the leading cause of ARF/RHD, Joseph et al. (2013) reported that a history of upper respiratory tract infection before the onset of the disease is the second most important risk factor, as 58% of RHD/ARF patients have a positive history [19]. Still, Ibrahim-Khalil reported a history of throat infection in 46% of RHD patients in Khartoum in 1992 [20]. In this study, 69.2% of RHD patients have a history of throat infection in the past six months; however, this was statistically significant only among patients whose mothers were unemployed. A larger sample size is needed to reach a more accurate assessment of this association.

Although ARF is a known prequel to RHD, Tadele (2013) reported that only 25% of the patients have a history of ARF, while Gopal (2012) found a history suggestive of ARF in 50% of Indian patients [21,22]. In our patient, a history of ARF was significantly associated with RHD (OR 60.8, 95% CI 10.3-356), as 82.1% of RHD patients have a history.

The rural residence is considered a risk factor for ARF/RHD. Locally, Alkhalifa in 2008 reported that 93% of patients are from rural and peri-urban areas [23], the same result concluded by Negi et al. (2013) conducted in Shimla, India [24]. These were observations, but they were insignificant when we controlled for residence.

The mother's education and employment status were explored as risk factors for ARF/RHD since mothers and their children are closely associated. In Bangladesh, Riaz et al. (2013) found that children of working or illiterate mothers were more likely to have the condition [18]. Furthermore, Dobson et al. (2011) found a strong correlation between RHD and mother unemployment in Fiji [25]. However, this study found no connection, which may be explained by the low level of education that Sudanese mothers generally possess.

Regarding regional distribution, most patients in an earlier study came from western states (Kordofan and Darfur) [12]. This matches our data, which shows that these states accounted for 41% of the patients; the Blue Nile state came in second with 38.5% of RHD cases. It has been found that RHD is concentrated in particular regions; these results can be used to guide the development of control programs.

## Conclusions

Our findings indicate a substantial correlation between RHD and an ARF history. There appears to be a correlation between residing in western states and sore throats. The findings of this study may provide valuable insights for the implementation of RHD control initiatives in Sudan.
